# Processing and Characterization of a Novel Distributed Strain Sensor Using Carbon Nanotube-Based Nonwoven Composites

**DOI:** 10.3390/s150717728

**Published:** 2015-07-21

**Authors:** Hongbo Dai, Erik T. Thostenson, Thomas Schumacher

**Affiliations:** 1 Civil and Environmental Engineering, University of Delaware, Newark, DE 19716, USA; E-Mail: hongbo@udel.edu; 2 Mechanical Engineering and Materials Science & Engineering, University of Delaware, Newark, DE 19716, USA; E-Mail: thosten@udel.edu; 3 Center for Composite Materials, University of Delaware, Newark, DE 19716, USA

**Keywords:** Carbon nanotubes, distributed sensing, structural health monitoring, nanocomposites, strain sensors, longitudinal and transverse sensitivity, civil infrastructure

## Abstract

This paper describes the development of an innovative carbon nanotube-based non-woven composite sensor that can be tailored for strain sensing properties and potentially offers a reliable and cost-effective sensing option for structural health monitoring (SHM). This novel strain sensor is fabricated using a readily scalable process of coating Carbon nanotubes (CNT) onto a nonwoven carrier fabric to form an electrically-isotropic conductive network. Epoxy is then infused into the CNT-modified fabric to form a free-standing nanocomposite strain sensor. By measuring the changes in the electrical properties of the sensing composite the deformation can be measured in real-time. The sensors are repeatable and linear up to 0.4% strain. Highest elastic strain gage factors of 1.9 and 4.0 have been achieved in the longitudinal and transverse direction, respectively. Although the longitudinal gage factor of the newly formed nanocomposite sensor is close to some metallic foil strain gages, the proposed sensing methodology offers spatial coverage, manufacturing customizability, distributed sensing capability as well as transverse sensitivity.

## 1. Introduction

All structural systems, including civil, mechanical, and aerospace structures, undergo aging and deterioration while in-service. In order to maintain their integrity and reliability, structural health monitoring (SHM) is increasingly important. Although a variety of sensors, such as strain gages and accelerometers, have been widely employed in SHM systems, most sensors only provide measurements at discrete locations or in a fixed direction on the structure [1]. Point sensors, such as strain gages, will often not be sensitive to localized damage [1,2]. Accurate assessment of structural health often requires dense arrays of sensors on critical members. In addition, field applications of traditional metallic-foil strain gages always suffer from performance degradation over time owing to temperature, moisture effects, and wiring problems [1,3]. As a result, there is a critical need to develop new distributed sensors that can monitor structural health consistently and reliably.

The emergence of nanotechnology has enabled engineers to tailor the properties of new materials at the nanoscale [4]. In particular, as an active nanoscale material modifier, Carbon nanotubes (CNT) are extremely small in size and possess excellent properties in terms of stiffness, strength, and electrical and thermal conductivities [5]. It has been demonstrated that by properly integrating CNTs into conventional composites, engineers are able to tailor and enhance their mechanical and physical properties [4,6]. CNT-based composites have been extensively investigated due to their excellent mechanical [4,7,8,9,10], electrical [11,12,13], and thermal [14,15,16] properties. In addition, CNTs have the ability to impart multi-functionality to existing composite material systems by enabling the simultaneous tailoring of different properties [4,5,17,18]. Especially, the strain sensing functionality of CNT-based composites has been studied experimentally [19,20,21,22,23,24,25] and investigated with numerical simulations [26,27,28,29,30]. Under an applied strain, CNT-based composites are piezoresistive where the mechanical deformation is coupled to changes in bulk resistivity of the composite. The piezoresistive response for these nanocomposites are mainly attributed to two aspects, including: (1) loss of nanotube-to-nanotube contact and separation of tunneling gaps resulting from deformation and reconfiguration of the conductive networks [6,7,19,20,22,24,31,32], and (2) piezoresistivity of CNTs themselves, as load is transferred to the nanotube via the interface of the polymer [21,23,33]. It has been shown that by measuring the overall electrical resistance change in the strained CNT-based nanocomposites the strain, as well as the onset, nature, and evolution of damage in the nanocomposites, can be quantified and monitored in real-time [2,18,34,35,36,37], which shows the potential for offering SHM functionality.

With this unique piezoresistive sensing response [19,32,38,39,40], CNT-based composites can be utilized as *in situ* strain sensors when integrated directly into the structural material [2,35,36,41,42] or as *ex situ* sensors that can be attached to a structure (*i.e.*, strain sensors [19,20,21,22,23,24] or body motion sensors [43,44]). For instance, Dharap *et al.* [21,45] utilized CNT assemblies formed into a macroscopic sheet, often referred to as “buckypaper”, and observed a nearly linear trend in the film resistance response when subjected to tensile and compressive cycles within ±0.02% strain. Similarly, Kang *et al.* [23] systematically characterized the piezoresistive responses of buckypaper and CNT/poly methyl methacrylate (PMMA) composites under static and dynamic loading conditions and demonstrated linear piezoresistivity up to 0.05% and 0.13% strain, respectively; the reported CNT/PMMA sensors showed gage factors (GF) of 1.0 to 5.0 and they also produced a long continuous strain sensor and suggested potential SHM applications. Additionally, Park *et al.* [19] developed CNT/polyethylene oxide (PEO) composite strain sensors and tested them in tension. In the linear response region of strain less than 0.6%, CNT/PEO sensors demonstrated GF of 1.6 and 3.7 for 1.44 vol.% and 0.56 vol.% CNT loading, respectively. Loh *et al.* [25,38] showed that CNT/polyelectrolyte thin films fabricated using a layer-by-layer process can be used for strain sensing with a linearity up to 1.0% strain and GF from 0.1 to 1.8. Especially, Hu *et al.* [32] performed a parametric study on sensor sensitivity in CNT/epoxy composite strain sensors and reported bilinear piezoresistive responses due to compression and tension loads. The highest GF of 7.0 in compression and 22.4 in tension were achieved within ±0.6% strain. All of these CNT-based sensors are formed with blending raw nanotubes at a relatively high concentration, making it difficult and costly to scale-up for monitoring of large structures.

Thostenson and Chou [18] demonstrated the capability of CNTs to form sensing networks around structural fibers and their subsequent use as *in situ* sensors. They created an electrically percolating nanotube network within a conventional glass fiber/epoxy composite by infusing epoxy resin with dispersed Carbon nanotubes into unidirectional glass fiber mats. The experimental results demonstrated the electrical/mechanical response measured in real-time as monotonically-loaded in tension. The specimen shows linear piezoresistivity at small strains and shows jumps in electrical resistance corresponding to the initiation of micro-scale cracks. Their work demonstrated the ability to sense strain as well as the onset and accumulation of micro-scale damage occurring within the composite. Additional experimental studies utilizing CNTs as *in situ* sensors have been conducted by Thostenson and Chou [41] and Gao *et al.* [34,35,36,37] for detecting damage in composite systems. One major limitation of this approach is that the CNTs need to be dispersed into the polymer, which is time consuming. In addition, the polymer experiences a sharp increase in viscosity with the addition of CNTs, making subsequent processing of large-scale sensors difficult. Finally, Schumacher and Thostenson [2], Ulbertini *et al.* [46] and Saafi [47] have studied CNT-based *in situ* sensors for use in civil structures.

Motivated by our preliminary work [48], this study introduces a simple two-step method to fabricate CNT-based nonwoven composite strain sensors where CNTs are deposited from an aqueous solution onto a selected nonwoven carrier fabric, followed by infusing an epoxy resin into the CNT-modified nonwoven fabric. This manufacturing approach can be readily scaled up for large-scale monitoring. In addition, the sensor utilizes a relatively small concentration of CNTs of approximately 1.0% by weight, making it cost-effective. The as-fabricated nanocomposite sensor is mechanically robust, strain sensitive, and customizable in shape, which is especially important for SHM of large-scale structural members. By depositing CNTs onto the preselected aramid nonwoven carrier fabric, an electrically-isotropic nanotube network is formed on the surface of the fibers. Epoxy is then infused into the fabric in order to hold the CNT network in place. The mechanical and piezoresistive response of sensors produced with different nanotube concentrations were studied. After characterizing the *in situ* strain sensitivity of the nonwoven sensing composite, the proposed sensors were bonded onto metal substrates, including aluminum and steel, and tested under quasi-static cyclic tensile and compressive loads.

## 2. Sensor Manufacturing and Characterization

The sensing approach involves the formation of the CNT network onto a carrier fabric. This approach enables application flexibility, since the fabric can be conformed to a variety of substrate configurations. Nonwoven fabrics composed of randomly oriented short chopped fibers were chosen as the carrier since the random fiber orientation results in in-plane isotropic electrical conductivity. A preliminary study of different candidate carrier fabrics with different types of fibers and areal weights demonstrated that an aramid nonwoven fabric (26 g/m^2^, Technical Fiber Products, Inc., Schenectady, NY, USA) showed the highest repeatability and best stability with respect to resistance-strain response. The CNT aramid nonwoven composite sensors were utilized for the comprehensive investigation presented in this paper.

### 2.1. Materials and CNT Composite Sensor Manufacturing

**Figure 1:**
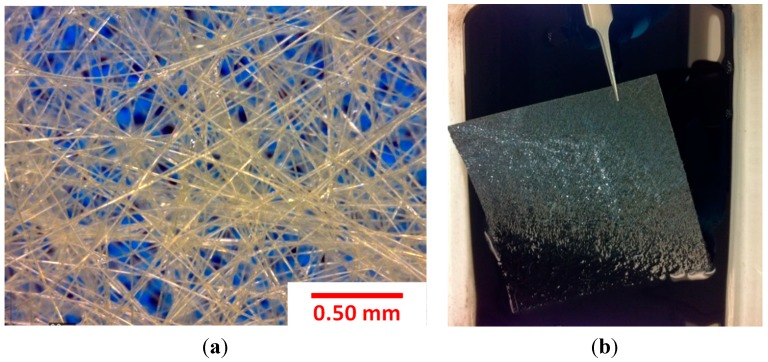
Photographs showing (**a**) porous fiber structure of the selected aramid nonwoven fabric (with porosity about 90%) and (**b**) a saturated aramid nonwoven fabric with CNTs following bath impregnation.

By depositing nanotubes onto the porous nonwoven fabric a macroscopic nanotube sensor can be produced with a relatively small concentration of CNTs. Figure 1a shows the fiber structure of the selected aramid nonwoven fabric that has an approximate void fraction of 90%. The CNT coating on the carrier aramid fabric is achieved using a solution casting process. The solution is a commercially available fiber sizing agent where nanotubes are dispersed in an aqueous solution with other polymers (SIZICYLTM XC R2G, Nanocyl). The overall CNT concentration in the sizing agent was found to be approximately 1.5% by weight (wt.) based on thermogravimetric analysis of the solids after drying. Prior to coating the fabric, the sizing agent was diluted with ultra-pure distilled water at mass ratios of 1:1 and 1:2 (sizing:distilled water by mass) and result in a nanotube concentration of 0.75 wt % and 0.5 wt %, respectively, in the as-prepared solutions for the CNT coating process. To ensure a uniformly dispersed solution, the diluted sizing was first mixed using a centrifugal mixer (THINKY^®^ ARM-310) at 2000 rpm for 120 s and then sonicated for 15 min in an ultrasonic bath (Branson^®^ 1510). Finally, the fabric was dipped for 20 min into a 1-liter flat-bottom glass container containing 100 mL of sizing solution. Figure 1b shows a fabric saturated with the carbon nanotube solution.

After drying the saturated fabric at 130 °C, the CNTs deposited onto the fabric form an electrically-conductive network on the fiber surface. An epoxy resin was infused into the fabric to form a free-standing sensing composite where the nanotube network is protected by the polymer matrix. The vacuum-assisted resin transfer molding (VARTM) technique was used to produce the sensing composite.

**Figure 2:**
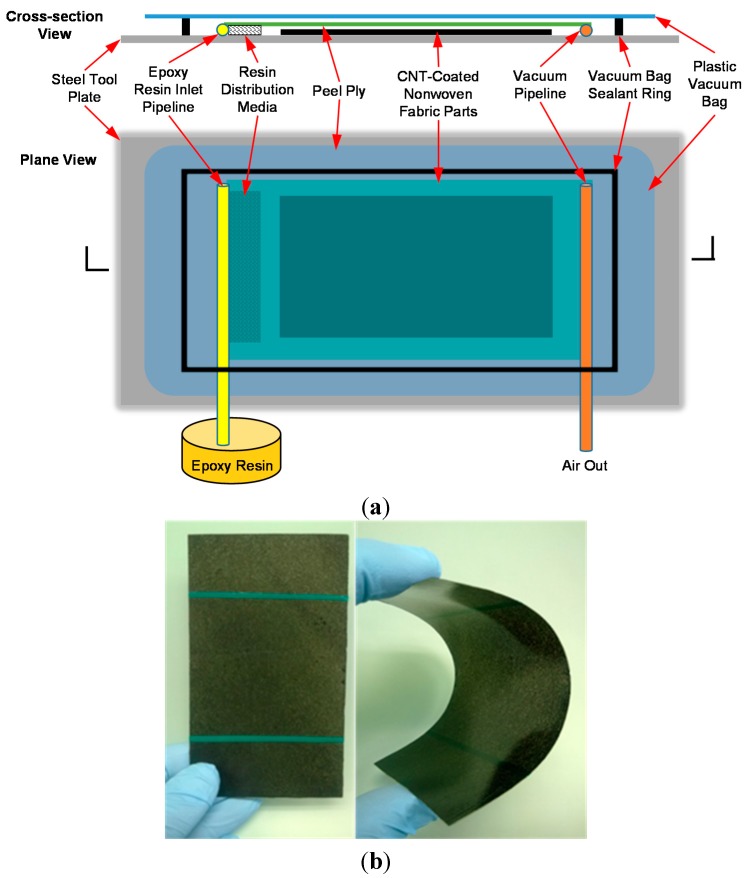
(**a**) Schematic diagram of the VARTM process utilized to infuse epoxy resin into the nonwoven fabric to form the CNT composite sensors and (**b**) photograph showing a free-standing CNT sensing composite after curing the epoxy, showing its flexibility.

The epoxy resin (EPON^®^ 862, Momentive Specialty Chemicals, Columbus, OH, USA) was first mixed with an aromatic diamine curing agent (EPIKURE W, Momentive Specialty Chemicals) and degassed at 60 °C for 20 min in a vacuum oven. The resin was then infused into the fabric using the VARTM setup as illustrated in Figure 2a. After completing the resin infusion, the epoxy was cured in the oven at 130 °C for 6 h. Figure 2b shows an as-produced sensing composite. The final lamina thickness is approximately 470 µm. Figure 2b also illustrates the flexibility of the sensor and its ability to conform to the shape of structural members. CNT composite sensors have also been produced using a room temperature curing agent (EPIKURE 3223, Momentive Specialty Chemicals) allowing the fabric to conform to the surface of the structure. In addition, the sensing composites have a very low fiber volume fraction, approximately 8%, owing to the high porosity of the nonwoven fabric. Based on the calculated concentration of Carbon nanotubes in the sizing and the mass change of the fabric after coating, it is estimated that the total concentration of nanotubes in the sensors are 1.0 wt % and 0.75 wt % for sizing dilution ratios of 1:1 and 1:2, respectively.

### 2.2. Sensor Microstructure Characterization

In order to study the structure of the electrically-conductive nanotube network, scanning electron microscopy (SEM) was utilized to image the morphology of the CNT coating on the aramid nonwoven fabric, as well as image fracture surfaces of the as-produced composite sensors. The specimens were imaged with an AURIGA™ 60 Crossbeam™ FIB-SEM with a 5 kV acceleration voltage. To minimize sample charging, all three samples were coated with a thin conductive Pt/Au layer (~5 nm) in a vacuum sputter coater (Denton Desk IV, Denton Vacuum, LLC) prior to imaging.

### 2.3. Mechanical and Electrical Characterization

To fully characterize the sensing response of the as-manufactured CNT composite sensors, a series of simultaneous mechanical and electrical measurements were conducted under tensile and compressive loading conditions. Prior to conducting electrical measurements, electrodes were applied using conductive silver paint (SPI Flash-Dry™, Structure Probe Inc., West Chester, PA, USA) and wires were anchored to the electrodes using conductive epoxy resin (EPOXIES^®^ 40-3900, Epoxies, Etc, Cranston, RI, USA). On the other face of each specimen, a 350 Ω strain gage (Micro-Measurements^®^, Vishay Intertechnology, Inc., Malvern, PA, USA) with a 3.2 mm gage length was bonded at the center of the specimen to measure the applied strain. The mechanical characterization was then conducted while simultaneously measuring electrical property changes. The mechanical loading protocols for the various specimens are discussed in the following sections. Electrical measurements of the sensors were conducted and synchronized with the applied loading protocols in real-time. A voltage-current meter (Keithley 6430 Sub-Femtoamp Remote SourceMeter, Keithley Instruments, Inc., Cleveland, OH, USA) was used to measure the electrical resistance of the sensors by sourcing a constant voltage of 10 V and measuring the resulting current to calculate the electrical resistance. All of the measurements, including load, strain, and electrical resistance, were controlled and collected using a customized LabVIEW program (LabVIEW 8.5, National Instruments Corporation, Austin, TX, USA). When multiple sensors were measured simultaneously, such as those described in Section 2.3.2, the lead wires were connected to a multiplexer (Keithley 3706A System Switch/Multimeter, Keithley Instruments, Inc., Cleveland, OH, USA). This multiplexer was linked with the voltage-current meter to rapidly switch between the four individual sensors.

#### 2.3.1. Mechanical/Electrical Response of the CNT Composite Sensors

**Figure 3:**
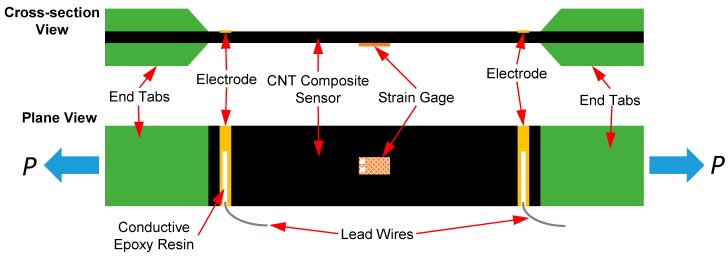
Schematic diagram of *in situ* CNT composite sensing specimens subjected to tension.

The mechanical and electrical response of the sensors were characterized in tension to determine their mechanical properties, the fundamental mechanical/electrical coupling response, and to establish the elastic limit of the sensor. Three sets of 76.2 × 127 mm sensing composites were prepared. One composite was made using only the aramid nonwoven fabric and tested as a reference; the other two composites were composed of the CNT-coated aramid nonwoven fabrics with CNT concentrations of 1.0 wt % and 0.75 wt % (per Section 2.1). All three sensing composites were cut into tensile specimens using a slot grinder equipped with a diamond blade. At least four test coupons were tested for each composite. For each coupon, a pair of 25.4 mm long nonconductive woven glass fiber/epoxy composite (G-10/FR4, Professional Plastics Inc., Fullerton, CA, USA) end tabs were bonded at each end using high strength epoxy paste adhesive (Hysol^®^ EA9309, Henkel). The electrodes are located at a distance of 3.2 mm from each edge of the end tab resulting in a gage length of 63.5 mm for all electrical measurements. The average electrical conductivities of the two sensing composites were measured as 4.7 S/m and 1.5 S/m for 1.0 wt % CNT and 0.75 wt % CNT composites, respectively. All specimens were subjected to monotonic tensile loading and tested to failure at a constant displacement rate of 1.3 mm/min using a screw-driven universal testing machine (Instron 5567, Instron, Norwood, MA, USA). Figure 3 illustrates the configuration of the CNT composite specimens. In particular, for the reference tension coupons, an acoustic emission (AE) system (Physical Acoustics Corporation, Princeton Junction, NJ, USA) was used to monitor micro-fractures to establish the elastic tensile strain limits of the specimens. An AE sensor with 35–100 kHz operating frequency was mounted on the center of the specimens. A threshold of 35 dB was selected to eliminate AE events due to environmental noise that were not related to the formation of damage in the specimen.

#### 2.3.2. Strain Monitoring of CNT Composite Sensors on Metallic Substrates

**Figure 4:**
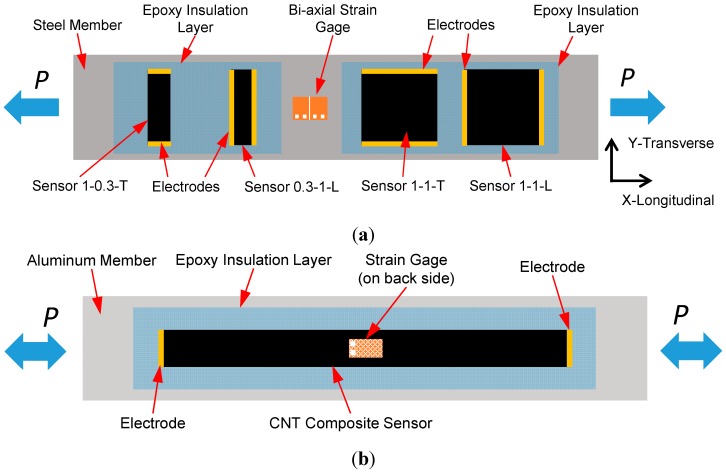
Schematic diagrams of test specimens for (**a**) longitudinal/transverse strain sensing on the steel substrate (Case Study 1) and (**b**) uniaxial tensile/compressive strain monitoring on the aluminum substrate (Case Study 2).

**Case Study 1**: Sensors on a steel bar—to investigate the sensing capability of the CNT composite sensors in the longitudinal and transverse directions, four sensors were distributed on a steel (ASTM A572 Grade 50) dogbone-shaped tensile bar (dimensions: 432 × 64 × 5 mm) with a neck area of 229 × 38 mm. These sensors are composed of the aramid nonwoven fabric processed with 1.0 wt % CNT loading and adhered along the longitudinal centerline of the steel bar using epoxy resin (EPON^®^ 862) with a curing agent (EPIKURE 3223). In accordance with the sensor size as well as the electrical current sourcing direction between the electrodes, these sensors are referred to as Sensor 1-1-L, 1-1-T, 0.3-1-L and 1-0.3-T as shown in Figure 4a. Specifically, Sensor 1-1-L and Sensor 1-1-T are 25.4 × 25.4 mm (*i.e.*, aspect ratio = 1) and their electrical measurements are made in the longitudinal and transverse directions, respectively. Similarly, Sensor 0.3-1-L and Sensor 1-0.3-T are 9.5 mm in the *x*-direction and 31.8 mm in the *y*-direction; they are monitoring the axial strain in the longitudinal direction and the transverse strain due to the Poisson contraction, respectively. A 350 Ω bi-axial stain gage (0°/90° pattern, Micro-Measurements^®^) with a gage length of 6.4 mm was used to measure the strains in both principal directions. The steel specimen was then subjected to a quasi-static loading protocol using an Instron 8562 (Instron, Norwood, MA, USA) servo-hydraulic load frame. The displacement-controlled loading protocol included five continuous load-unload cycles: two to 110 MPa and three to 248 MPa using a displacement rate of 0.5 mm/min and an unloading rate of 0.8 mm/min. During the second and fifth loading cycles, the stress was maintained for 30 s to examine any transient effects. The test was discontinued at a peak load corresponding to 300 MPa.

**Case Study 2**: Sensors on aluminum bars—to further verify strain monitoring capabilities of the proposed CNT-based strain sensors, two uniaxial strain monitoring specimens were further investigated on aluminum substrates. In the first specimen, a sensor size of 88.9 × 12.7 mm, composed of the aramid nonwoven fabric with CNT concentration of 1.0 wt %, was mounted at the center of a dogbone-shaped aluminum tensile bar (152 × 25 × 2 mm, 6061-T6). This specimen (Al-1.0%-CNT) was subjected to incremental tensile cyclic loadings performed using an Instron 5567 (Instron, Norwood, MA, USA) universal testing machine at a fixed displacement rate of 1.3 mm/min. The loading protocol consisted of six steps with load amplitudes of 23.5, 47.1, 70.6, 106, 141, and 188 MPa. A 47.1 MPa loading-unloading cycle was placed between the 106, 141, 188 MPa steps in order to validate the electrical stability of the sensing layer. In the second specimen, a 63.5 × 9.5 mm sensor containing 0.75 wt % CNT was bonded on a 6.4 mm thick aluminum bar (152 × 25.4 mm, 6061-T6). This specimen (Al-0.75%-CNT) was subjected to a seven-step compression-tension cyclic loading at the same displacement rate as specimen Al-1.0%-CNT. The initial loading cycle was ±24.8 MPa. Each loading step included two cycles with identical magnitude at the peak and a full compression-tension loading step with four individual cycles. The load steps increased from 24.8 to 99.2 MPa in tension at an even increment of 12.4 MPa and, due to the slenderness of this bar, the largest compressive load cycle was −62.0 MPa after the fourth compression step. A thin coating of EPON 862 epoxy resin bonds the sensor onto the substrate and also acts as an insulating layer. Figure 4b schematically shows the specimen configurations of Al-1.0%-CNT and Al-0.75%-CNT. Baseline electrical resistance measurements were collected on these two specimens and found to be 7.38 and 24.8 kΩ, respectively.

## 3. Results and Discussion

### 3.1. Sensing Composite Microstructure Characterization

**Figure 5:**
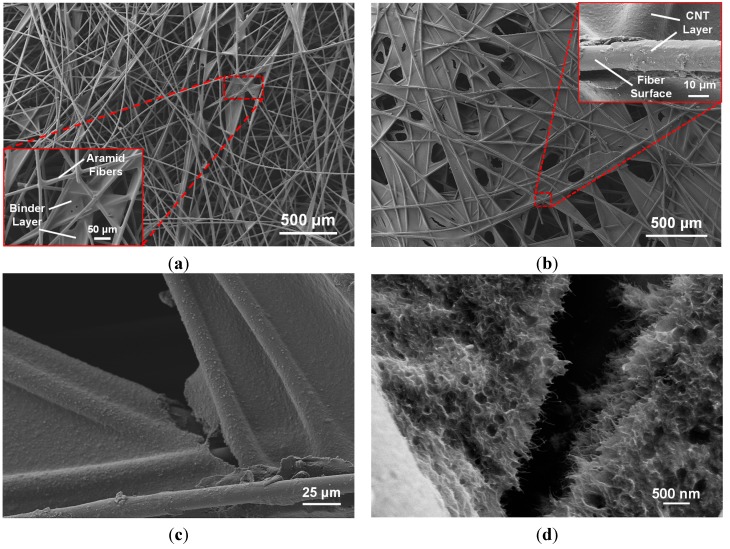
SEM micrographs showing (**a**) non-woven aramid fabric with binder (insert), (**b**) aramid fabric with CNTs deposited onto the fiber surface (inset), (**c**) fiber cross-over region with nanotubes uniformly coating the binder and fibers with (**d**) a high-magnification image showing the CNT coating.

Figure 5a shows the randomly distributed fiber architecture of the nonwoven aramid fabric in its as-received state. It can be seen that the binder creates a slightly rough layer on the fiber surface and the binder also accumulates in the cross-over regions, which further results in the increase of the fabric surface area.

As reported by the manufacturer, the binder is a cross-linked polyester in the amount of 12.5% by weight. Figure 5b shows the aramid fabric after the deposition of CNTs. There appears to be wettability between the nanotube sizing and the fibers. The polymer solids in the sizing act to further bind together the fibers and form the electrically-conductive network spanning the individual fibers. Locally there is some stripping of the nanotube coating, which is likely a consequence of handling the fabric after drying. Figure 5c shows the nanotube coating that formed at a typical fiber crossover region. There is cracking in the region of the crossover, also likely due to handling the fabric after drying, highlighting the need to further protect the fabric layer through infusion of an epoxy matrix. Figure 5d shows a high-magnification SEM image of the formation of a layer with a large concentration of CNTs. The large amount of nanotubes deposited on fiber surfaces effectively forms an electrically-conductive network over the entire fabric.

**Figure 6:**
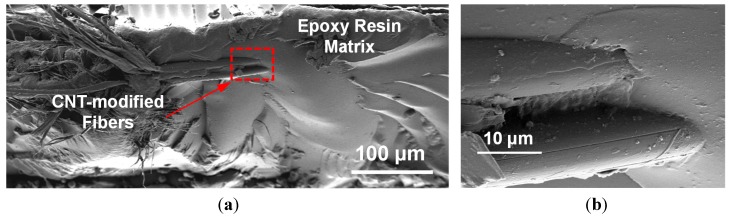
SEM image showing (**a**) fracture surface of nanocomposite sensor fabricated in this study and (**b**) a high-magnification image showing the CNT-modified fibers (dashed area in (a)).

Figure 6a shows a fracture surface of the fabric/epoxy composite after resin infusion where three fibers are seen protruding from the polymer matrix. It can be seen that the nanotube-based sensor has a low fiber volume fraction overall. From the higher-magnification view in the region of the nanotube-modified fibers in Figure 6b, strong wetting between the coated fibers and the polymer matrix is observed.

### 3.2. Mechanical and Electrical Characterization of CNT Composite Sensors

**Figure 7:**
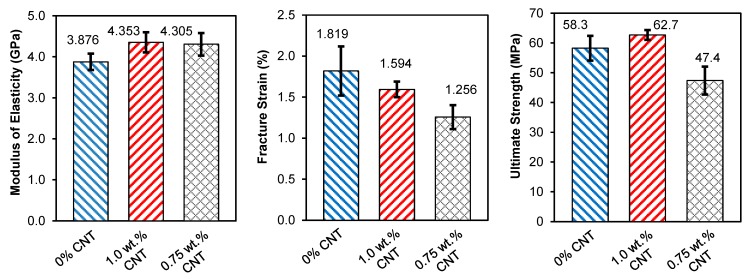
Mechanical properties of the aramid/epoxy composite (=0% CNT) and the CNT composite sensors (=1.0 and 0.75 wt %) (error bars represent one standard deviation).

The composites are composed of a relatively low volume fraction of fibers, approximately 8%, which are also randomly oriented, resulting in mechanical properties dominated by the epoxy. The composites, which include the aramid without the nanotube coating, as well as the 1.0 wt % and 0.75 wt % CNT loadings, all show linear stress-strain behavior. Based on acoustic emission (AE) measurements, it was noted that the acoustic activity increases shortly after 0.4% strain. This incipient damage is likely due to fiber/matrix interface debonding, as well as matrix microcracking. At higher stresses there is a significant increase in AE activity with a decrease in the slope of the stress-strain curve, corresponding to inelastic behavior due to the accumulation of cracks. Based on these observations, the elastic limit of the sensors was established at 0.4% strain. Figure 7 summarizes the mechanical properties of the different aramid/epoxy composites. With the integration of CNTs, there is a 12% increase in the elastic modulus with the CNT coatings. While there is an increase in stiffness, the coating makes the specimens more brittle, and the ultimate failure strain decreases with the addition of the CNT coating. This adverse effect is in agreement with the experimental study by Ci and Bai [49]. Although there is a decrease in failure strain there is a slight increase in strength for the sensing composite with 1.0 wt % CNT loading, owing to its higher elastic modulus. The composite sensors with 0.75 wt % CNT loading show a slight decrease in strength.

**Figure 8:**
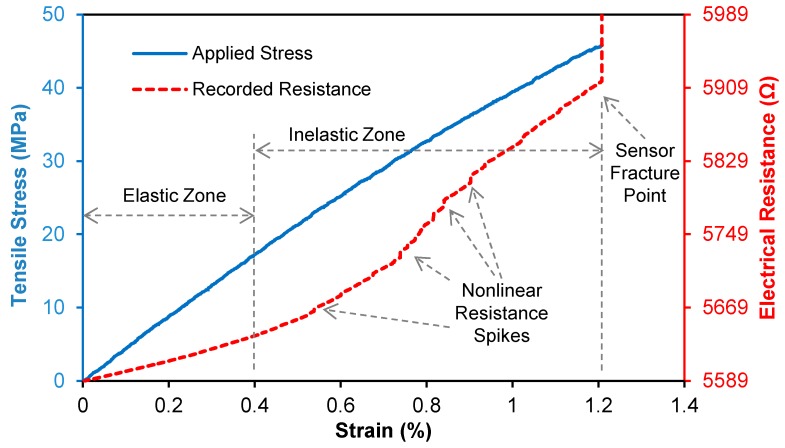
Typical stress and electrical resistance *vs.* strain responses of CNT composite sensors.

Figure 8 shows a typical stress and electrical resistance response with applied tensile strain for a CNT composite sensor. In the elastic zone, the resistance-strain behavior of the sensor is linear. At strains higher than the elastic zone spikes are observed in the resistance-strain curve and correspond the formation of cracks that permanently sever portions of the electrically conductive network. This progressive increase in microcracks results in a change in slope of the resistance-strain behavior and corresponds to the inelastic behavior of the sensors. The piezoresistive behavior of the nanotube sensors are quantified in terms of their gage factors (GF). In this study, we defined gage factors under two specific strain levels corresponding to the un-cracked elastic state (under 0.4% strain) and the matrix-cracked inelastic state (above 0.4% strain) by performing a linear least-squares curve-fit on the experimental data.

**Figure 9:**
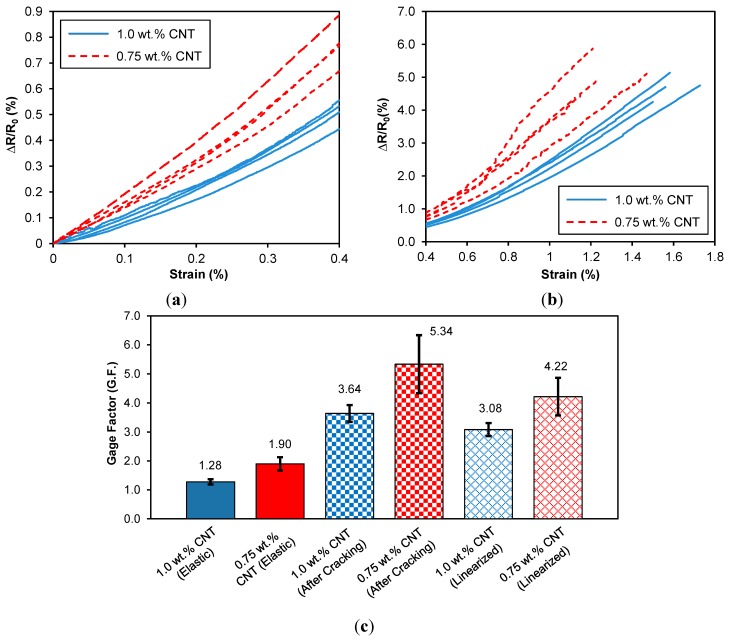
(**a**) Elastic and (**b**) inelastic piezoresistive responses from individual test coupons and (**c**) gage factors for the CNT-based nonwoven composite sensors fabricated in this research (error bars represent one standard deviation).

The resistance-strain responses of CNT composite sensors for the elastic state and inelastic states are shown in Figure 9a,b, respectively. Figure 9c summarizes gage factors obtained for both groups of specimens in accordance to the elastic and inelastic strain levels. It can be seen that the group of four specimens with 0.75 wt % CNT loading shows higher strain sensitivity than 1.0 wt % CNT group. There have been a number of experimental studies on the piezoresistive behavior of nanocomposites based on CNTs. Experimental results [19,20,24,29,32] indicate that there is a higher degree of sensitivity with reduced CNT concentration, and the general trend in gage factor is consistent with that reduction. However, the elastic gage factors are somewhat lower than expected as compared to nanocomposites with nanotubes dispersed throughout the polymer matrix developed by other researchers [29,32,33]. This lower gage factor is attributed to the formation of the conducting networks preferentially along the surfaces of the fibers. The high concentration of CNTs on the fiber surface results in a nanocomposite “interphase” that forms around the fiber. This interphase region effectively acts like an overall nanocomposite sensor that has a high volume fraction of nanotubes. In addition, it is likely that the random fiber architecture also influences the piezoresistive response. Meanwhile, the inelastic gage factors are also higher than the elastic gage factors. The breaking up of the CNT network due to cracking and fiber-matrix debonding severs conducting pathways, resulting in a lower effective volume fraction of nanotubes conducting current in the sensor.

### 3.3. Strain Monitoring of CNT Composite Sensors on Metallic Substrates

#### 3.3.1. Case Study 1: Longitudinal and Transverse Strain Sensing on a Steel Substrate

**Figure 10:**
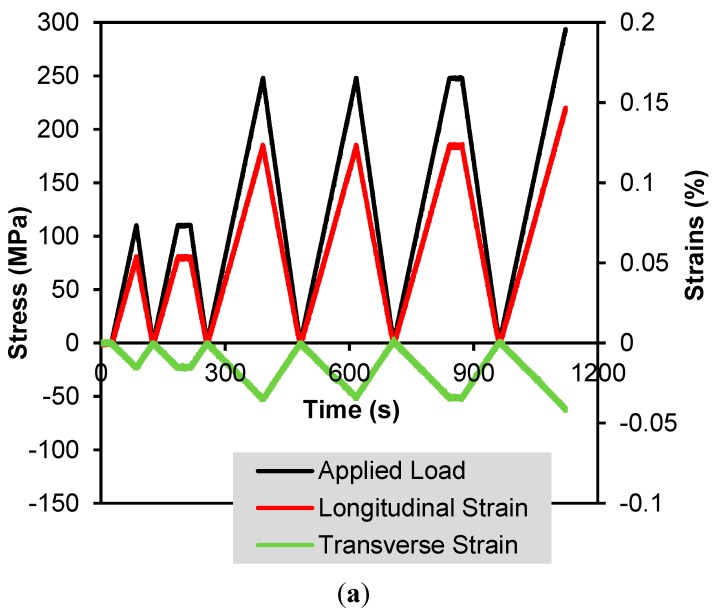

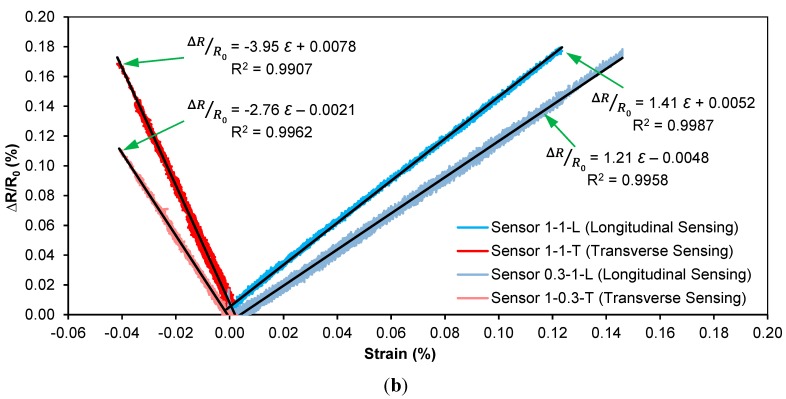
(**a**) Applied load and longitudinal and transverse strain of the steel specimen and (**b**) the sensing response of the four sensors, showing the linearity and gage factors estimated using linear least squares curve fitting.

Figure 10a shows the mechanical response of the steel specimen (Specimen 1) subjected to a longitudinal tensile stress as well as its transverse strain response due to the Poisson contraction. It can be seen that this specimen deforms elastically under the applied loading. The corresponding sensing responses of the four CNT composite sensors are displayed in Figure 10b. All show a strong linear response where the piezoresistivity of Sensors 1-1-T (GF = −3.95) and 1-0.3-T (GF = −2.76) in the transverse direction is higher compared to Sensors 1-1-L (GF = 1.41) and 0.3-1-L (GF = 1.21) in the longitudinal direction. Although the strain in the transverse direction is compressive, the resistance of the sensing increases, resulting in a negative gage factor. Unlike a traditional strain gage, the CNT network is random and shows sensitivity to both transverse and longitudinal strain. At the nanoscale there are changes in tunneling gaps associated with both transverse and tensile strains in the sensor. In this case the longitudinal strain is much higher than the transverse strain. As a consequence, the net increase in the electrical tunneling gaps is dominated by the strain in the longitudinal direction resulting in an increase in resistance in both directions. In addition, under the same strain field, longer conductive pathways along the longitudinal strain direction attract more changes in CNT-to-CNT tunneling gaps, which result in the higher strain sensitivity in Sensor 1-1-L than Sensor 0.3-1-L.

#### 3.3.2. Case Study 2: Uniaxial Strain Monitoring on Aluminum Substrates

**Figure 11:**
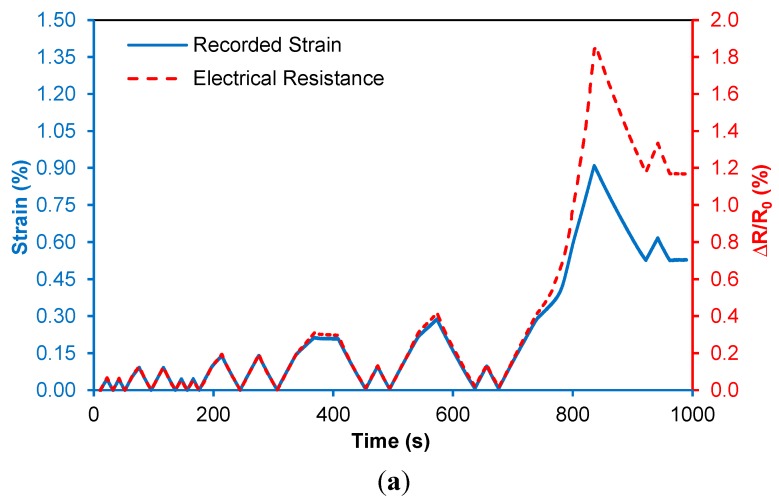

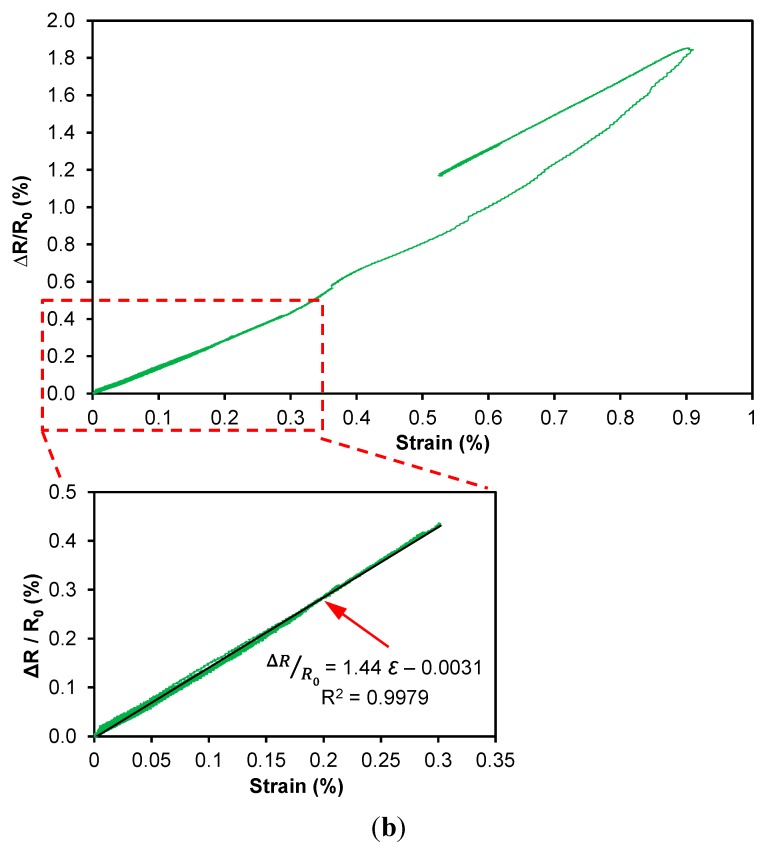
(**a**) Piezoresistive response of CNT-based composite sensing layer on Specimen Al-1.0%-CNT during the full cyclic loading test, showing the close tracking of strain and resistance up to plastic deformation of the specimen and (**b**) the resistance-strain response of the sensor (insert: elastic piezoresistive response).

**Specimen 1**: With the transverse sensitivity of the sensor established, understanding the uniaxial response of the sensor in tension and compression, as well as its response while the substrate undergoes plastic deformation, is important for SHM applications. Figure 11a shows the response of the sensor (Al-1.0%-CNT) due to applied tensile deformation. This specimen undergoes elastic deformation in the first 12 applied load cycles without permanent strain change. The response of the sensor tracks exactly with the elastic strain. Plastic deformation of the aluminum occurs beyond 0.3% strain, which is at 75% of the elastic limit of the sensor.

There is a permanent deformation of 0.52% strain at the end of this cycle. In addition to the plastic strain that occurs, this deformation is near the elastic limit of the CNT composite sensor, resulting in a larger sensitivity to the permanent deformation in the last cycle. Specifically, there is a permanent electrical resistance change of 1.2% (or 88 Ω in real resistance). It is clear that the trends in the resistance response of the attached composite sensor and strain measurements are very comparable and there are no resistance drifts observed in the electrical response of the sensor. Figure 11b shows the piezoresistive response of the CNT composite sensor during the full loading profile. As the specimen begins to deform plastically, resistance increases nonlinearly with strain and a permanent resistance change was found in the end of the loading corresponding to a strain of 0.52%. The evolution of the plastic deformation of the specimen is comprehensively reflected by the trend of the electrical resistance response, which is desirable. The insert of Figure 11b shows the linear elastic piezoresistive response corresponding to the first 12 applied elastic load cycles. It can be observed that under elastic loading, the behavior of the sensor (GF = 1.44) is quite linear compared to the strain gage measurement, which is promising.

**Figure 12:**
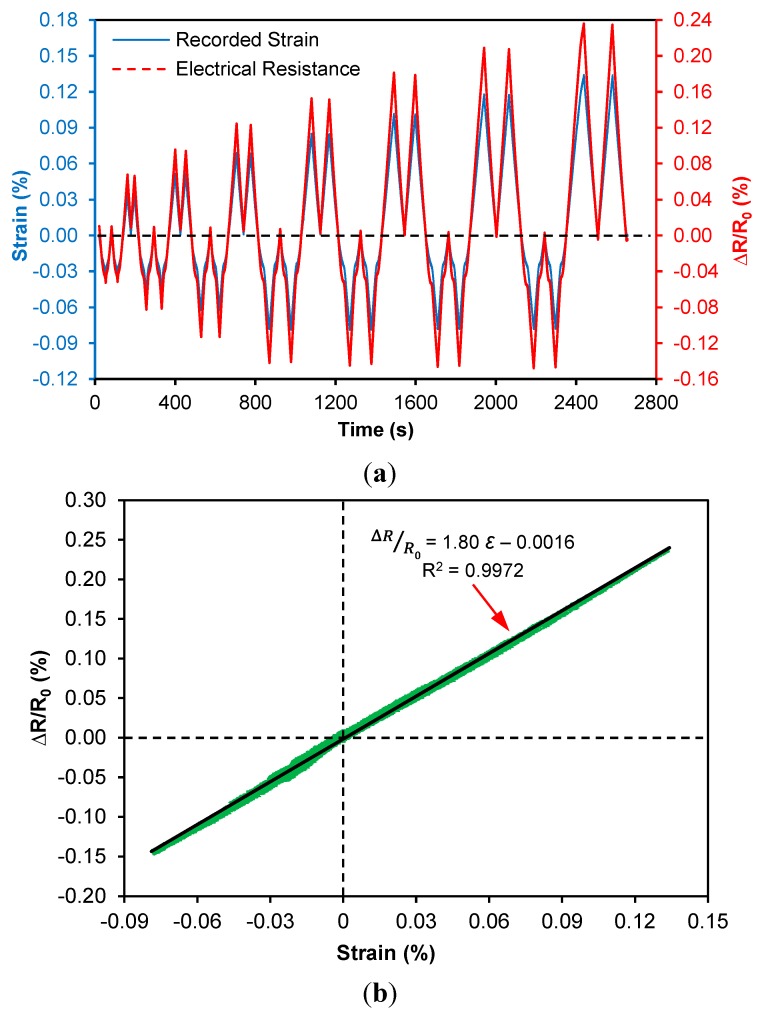
Linear-elastic piezoresistive response of CNT composite sensor on Specimen Al-0.75%-CNT according to applied compression-tension cyclic loads: (**a**) real-time response under cyclic loading and (**b**) linear piezoresistive behavior under tension-compression loading.

**Specimen 2**: For specimen Al-0.75%-CNT Figure 12a shows the tensile and compressive elastic piezoresistive response of the sensor under the prescribed loading cycle. The peak tensile strains are +0.14% and minimum compressive strains are −0.08%, which are all in the elastic range. Minimal change in recorded baseline resistance was anticipated between the transition between large and small loads. Under compression, the nanotube-nanotube tunneling gaps decrease resulting in more conductive pathways resulting in reduced electrical resistance of the CNT composite sensor. It can be seen that under the compression-tension cyclic loading protocol, the recorded electrical resistance displays very close correlations to the member strains. The sensor shows strong electrical stability as well as negligible amount of baseline resistance change among all compression-tension loading steps in real-time. From Figure 12b, it can also be seen that the piezoresistive response of the CNT composite sensor displays strong linearity for both compressive and tensile strains with a single gage factor, GF = 1.80 which is close to the gage factor (GF = 1.90) of the group of sensors with 0.75 wt % CNT loading characterized in Section 3.2, indicating a high degree of repeatability. These results are desirable and facilitate applications of this fabricated sensing layer for future SHM.

## 4. Conclusions

This research has established a simple and cost-effective approach for the manufacturing of novel carbon nanotube (CNT)-based piezoresistive composites that can be used as strain sensors for structural health monitoring (SHM) applications. This technique is readily scalable for field applications and has a high degree of application flexibility. We have successfully fabricated the nanotube-based strain sensors by, first, coating nanotubes onto the aramid nonwoven carrier fabric using a CNT-based fiber sizing agent, followed by infusing epoxy resin into the fabric to hold the nanotube network in place. Comparing with nanotube-based films (*i.e.*, buckypaper), the nonwoven composite sensors in this study have a much lower concentration of CNTs, which will substantially reduce cost and facilitate engineering applications. The sensors are mechanically robust and show a linear piezoresistive response. The mechanical and electrical properties of the as-manufactured CNT composite sensors have been characterized under quasi-static tensile tests. Linear piezoresistive responses with an elastic gage factor (GF) of 1.90 and a nonlinear GF of 5.34 corresponding to the longitudinal strains, have been obtained for these CNT composite sensors.

The transverse sensitivity of the sensor has also been established and shows negative piezoresistivity (with an elastic gage factor, GF = −3.95) in the transverse direction. In the last part of this study, two experiments of uniaxial quasi-static strain monitoring applications have been performed on metallic members, which were subjected to a quasi-static cyclic elastic and plastic tensile loading protocols and a compression-tension loading cycle, respectively. For the newly developed CNT composite sensors, strong linearity in the piezoresistive responses due to elastic tensile and compressive strains have been observed and the permanent electrical resistance change corresponding to plastic deformation has also been identified. In addition, the real-time sensing capacity of these sensors has been further verified.
